# Consolidation of Sleep-Dependent Appetitive Memory Is Mediated by a Sweet-Sensing Circuit

**DOI:** 10.1523/JNEUROSCI.0106-22.2022

**Published:** 2022-05-04

**Authors:** Nitin S. Chouhan, Amita Sehgal

**Affiliations:** ^1^Howard Hughes Medical Institute, Perelman School of Medicine at the University of Pennsylvania, Philadelphia, Pennsylvania 19104; ^2^Chronobiology and Sleep Institute, Perelman School of Medicine at the University of Pennsylvania, Philadelphia, Pennsylvania 19104

**Keywords:** appetitive memory, dopaminergic neurons, gustatory receptor neurons, sleep, sweet taste

## Abstract

Sleep is a universally conserved physiological state which contributes toward basic organismal functions, including cognitive operations such as learning and memory. Intriguingly, organisms can sometimes form memory even without sleep, such that *Drosophila* display sleep-dependent and sleep-independent memory in an olfactory appetitive training paradigm. Sleep-dependent memory can be elicited by the perception of sweet taste, and we now show that a mixed-sex population of flies maintained on sorbitol, a tasteless but nutritive substance, do not require sleep for memory consolidation. Consistent with this, silencing sugar-sensing gustatory receptor neurons in fed flies triggers a switch to sleep-independent memory consolidation, whereas activating sugar-sensing gustatory receptor neurons results in the formation of sleep-dependent memory in starved flies. Sleep-dependent and sleep-independent memory relies on distinct subsets of reward signaling protocerebral anterior medial dopaminergic neurons (PAM DANs) such that PAM-β′2mp DANs mediate memory in fed flies whereas PAM-α1 DANs are required in starved flies. Correspondingly, we observed a feeding-dependent calcium increase in PAM-β′2mp DANs, but not in PAM-α1 DANs. Following training, the presence of sweet sugars recruits PAM-β′2mp DANs, whereas tasteless medium increases calcium in PAM-α1 DANs. Together, this work identifies mechanistic underpinnings of sleep-dependent memory consolidation, in particular demonstrating a role for the processing of sweet taste reward signals.

**SIGNIFICANCE STATEMENT** Sleep is essential for encoding and consolidating memories, but animals must often suppress sleep for survival. Consequently, *Drosophila* have evolved sleep-independent consolidation that allows retention of essential information without sleep. In the presence of food, sleep is required for memory, but mechanisms that transmit signals from food cues to regulate the need for sleep in memory are largely unknown. We found that sweet-sensing neurons drive the recruitment of specific reward signaling dopaminergic neurons to establish sleep-dependent memory. Conversely, in the absence of a sweet stimulus, different neurons are activated within the same dopaminergic cluster for sleep-independent memory consolidation. Therefore, the processing of sleep-dependent memory relies on the presence of sweet sugars that signal through reward circuitry.

## Introduction

Sleep is a natural and reversible state of reduced responsiveness that serves an important role in cognitive functions, such as learning and memory. Experimental evidence for this, showing that sleep disturbances affect memory acquisition and retention, comes from humans, rodents, birds, and insects (Stickgold, 2005; Diekelmann and Born, 2010; Rasch and Born, 2013). However, sleep is influenced by environmental factors, some of which can induce extended periods of wakefulness. While these might be expected to impair memory, elephants, whales, and dolphins can actually learn and retain essential information even while behaviorally active for prolonged periods of time (Lyamin et al., 2005; Branstetter et al., 2012; Gravett et al., 2017; Siegel, 2021). The extent to which unihemsipheric sleep (sleep with one-half of their brain) contributes to the function of dolphins and whales under these conditions is controversial (Branstetter et al., 2012; Siegel, 2021).

The fruit fly, *Drosophila melanogaster,* has emerged as a valuable model to address mechanistic underpinnings of sleep, and it is also widely used to understand the cellular and molecular basis of learning and memory. Flies can form robust long-term associations between a neutral stimulus, such as an odor, and a sugar reward after only a single cycle of training (Krashes and Waddell, 2008). Intriguingly, flies fed after training need sleep to form long-term memory, but sleep is dispensable for memory consolidation in flies that are starved, and thereby induced to forage (Chouhan et al., 2021). Sleep-dependent and -independent memory map to mushroom bodies (MBs), the site of learning and memory in the fly brain, but to anatomically distinct subsets of α′/β′ MB lobes and to distinct recurrent circuits that link PPL1 dopaminergic neurons to corresponding MB output neurons (MBONs) (Chouhan et al., 2021). However, how flies switch between sleep-dependent and sleep-independent memory consolidation is not well understood.

Sweet taste acts as a reward in flies. Flies presented with a non-nutritive sweetener, such as arabinose or sucralose, and an odor form a short-term appetitive memory for the odor, while pairing of an odor with a tasteless nutritive substance, such as sorbitol, is not as effective as with a sweet-tasting sugar (Burke and Waddell, 2011; Fujita and Tanimura, 2011). Sweet taste is also sufficient to reduce foraging and promote sleep in starved flies (Yang et al., 2015; Hasegawa et al., 2017). Thus, flies rely on sweet taste to predict food availability (Stafford et al., 2012).

In the present study, we show that sweet taste, but not nutritional value, is necessary for sleep-dependent memory in an appetitive conditioning paradigm. Consistently, manipulation of sugar-sensing gustatory receptor neurons (GRNs) is sufficient to alter the need for sleep in memory consolidation in fed and starved flies. As reward signals in flies are mediated by PAM dopaminergic neurons (DANs) (Liu et al., 2012; Huetteroth et al., 2015; Yamagata et al., 2015; Tsao et al., 2018; Musso et al., 2019), we considered them as candidates for processing sweet taste signals relevant for memory, and show that sweet taste recruits a distinct subset of PAM DANs, PAM-β′2mp, for sleep-dependent memory consolidation. On the other hand, sleep-independent memory is mediated by PAM-α1 DANs, which are activated by nonsweet substrates following training. Together, we propose that the processing of sweet taste reward signals determines the requirement for sleep in memory consolidation.

## Materials and Methods

### Fly stocks and husbandry

Flies were reared on standard cornmeal fly food at 25°C and 60% relative humidity under a 12 h:12 h light:dark cycle. A randomized but aged-matched population of flies were transferred to fresh food vials 48 h before each trial. For starvation, flies were kept in empty bottles with wet cotton as a source of water. The background control line was Canton-S (Heisenberg) strain. The following fly lines were ordered from Bloomington stock center: 20XUAS-TTS-shi[ts1]-p10 (66600; referred to as UAS-*shibire^ts1^* in the text), UAS-*TrpA1* (26263), Gr64f-Gal4 (57669), R58E02 (41347), MB196B (68271), MB056B (68276), and MB299B (68310). UAS-*CaLexA* line was described previously (Masuyama et al., 2012).

### Appetitive conditioning assay

Appetitive conditioning was performed as described previously (Krashes and Waddell, 2008; Colomb et al., 2009). In brief, a mixed-sex group of ∼100 flies, 4-7 d old, was first starved for 18-20 h and then trained at 25°C and 70% relative humidity. During conditioning, flies were first presented with odor A conditioned stimulus (CS^–^) in an air stream with a water-soaked filter paper (blank) for 2 min. A 30 s stream of clean air was then followed by a presentation of sucrose unconditioned stimulus (US) with odor B (CS^+^) for 2 min. A filter paper soaked in 1.5 m sucrose solution and then dried was used as a US reward. In reciprocal experiments, Odor B was presented with blank, while Odor A was presented with sucrose. Odors, 4-methylcyclohexanol and 3-octanol, were diluted in paraffin oil at 1:10 concentration and were presented in 5 mm (4-methylcyclohexanol) and 3 mm (3-octanol) diameter cups in the air stream. Fed flies were moved to standard fly food, while starved flies were maintained in empty bottles after training. As starvation is necessary for memory retrieval (Krashes and Waddell, 2008), fed flies were restarved for 48 h before memory tests. To assess the role of sweet taste in memory consolidation, flies were moved to either 300 mm arabinose or 300 mm sucralose in 1% agar after training. Flies were kept on 300 mm sorbitol in 1% agar to test the effect of nutrient availability after training on memory consolidation. Flies kept on sucralose or sorbitol were tested 24 h after training for memory. Flies were sleep-deprived for the first 6 h following training with a mechanical stimulus using a mounting plate of Trikinetics vortexer, which involves horizontal shaking of fly vials for 2 s after every 20 s time interval.

To test for memory, flies were given a choice between Odor A and Odor B for 2 min in a T-maze. Performance index (PI) was measured as the number of flies selecting CS^+^ odor minus the number of flies selecting CS^–^ odor divided by the total number of flies. To minimize nonassociative effects, each PI is the average of PIs from reciprocal experiments with Odor A and Odor B swapped.

For experiments involving UAS-*shibire^ts1^* or UAS-*TrpA1* genotypes, flies were raised and starved at 22°C. Starved flies were trained at 23°C and 70% relative humidity and then moved to 32°C (restrictive temperature) for *shibire^ts1^* based silencing of neurons. UAS-*TrpA1* flies were kept at 22°C throughout experiments and only moved to 30°C for temperature-based induction.

### Sleep assessment

A mixed population of 50 male and 50 female flies were first trained, and then 32 flies from this group were introduced in 65 mm glass tubes through an aspirator without anesthesia and loaded into *Drosophila* activity monitors (DAMs, Trikinetics system). Flies presented with only sucrose without odor served as untrained controls. Flies were trained at Zeitgeber (ZT6), and sleep was measured from ZT8 onward because of the time spent in introducing flies into individual tubes and, also, to minimize the effects of handling on sleep. Locomotor data were collected using DAMsystem3 software, and raw data files were analyzed with DAMfilescan111. Five minutes of inactivity, defined as no beam breaks in the DAM, was classified as sleep (Hendricks et al., 2000; Shaw et al., 2000). Insomniac 3.0 was used to assess sleep data (Lenz et al., 2015).

UAS-*shibire^ts1^* flies were first trained at 23°C and then moved to 32°C to test the effect of silencing Gr64f^+^ neurons on sleep after training.

### Imaging

A standard protocol was used for fixation and staining. In brief, adult fly brains were first dissected in cold phosphate buffered saline (PBS) and then fixed in 4% paraformaldehyde (PFA) (v/v) for 20-30 min at room temperature. The brains then underwent three 15 min washes in PBS-0.3% Triton-X (PBST). Samples were then incubated in 5% normal goat serum in PBST (NGST) for 1 h and then incubated overnight with primary antibodies in NGST at 4°C. The next day, samples were rinsed in PBST 3 times 15 min each followed by incubation with secondary antibodies for 2 h at room temperature in NGST buffer. Another three 15 min washes were performed, and then brains were moved into 50% glycerol. An anti-fade medium (Vectashield: H1000) was used to mount brains on slides, and then brains were visualized on a Leica Microsystems TCS SP5 confocal microscope. Primary antibodies used are as follows: rabbit anti-GFP (1:1000; Thermo Fisher Scientific; catalog #A11122) and rat anti-RFP (1:1000; ChromoTek; catalog #5F8). Secondary antibodies used are as follows: AlexaFluor-488 goat anti-rabbit (1:1000; Thermo Fisher Scientific; catalog #A11008) and AlexaFluor-594 goat anti-rat (1:1000; Thermo Fisher Scientific; catalog #A11007). Rabbit anti-GFP primary with secondary AlexaFluor-488 goat anti-rabbit antibodies were used to detect GFP signal from the CaLexA (Calcium-dependent nuclear import of LexA) reporter system. Fiji 2.0 was used for analyzing images.

### Experimental design and statistical analysis

All statistical analyses were performed using GraphPad Prism 8.0 and are detailed in Results. D'Agostino and Pearson's omnibus test was used to test for normality. A two-sided Student's *t* test for two groups and one-factor ANOVA followed by Tukey *post hoc* test in the case of multiple groups were used for analyzing normally distributed data. Also, differences in sleep after training between trained and untrained groups were assessed using multiple *t* tests with Bonferroni correction. The data with non-Gaussian distribution were analyzed with a Mann–Whitney *U* test. The sample size is based on previous similar studies and is depicted in respective figures. The mean and standard error (SE) were used to represent the data. Individual data points are displayed as dots. In memory experiments, each data point represents a group of flies, and single fly data are depicted in sleep and imaging experiments. Group means are displayed in figures depicting sleep trends.

## Results

### Sweet taste drives sleep-dependent memory consolidation

**Figure 1.**
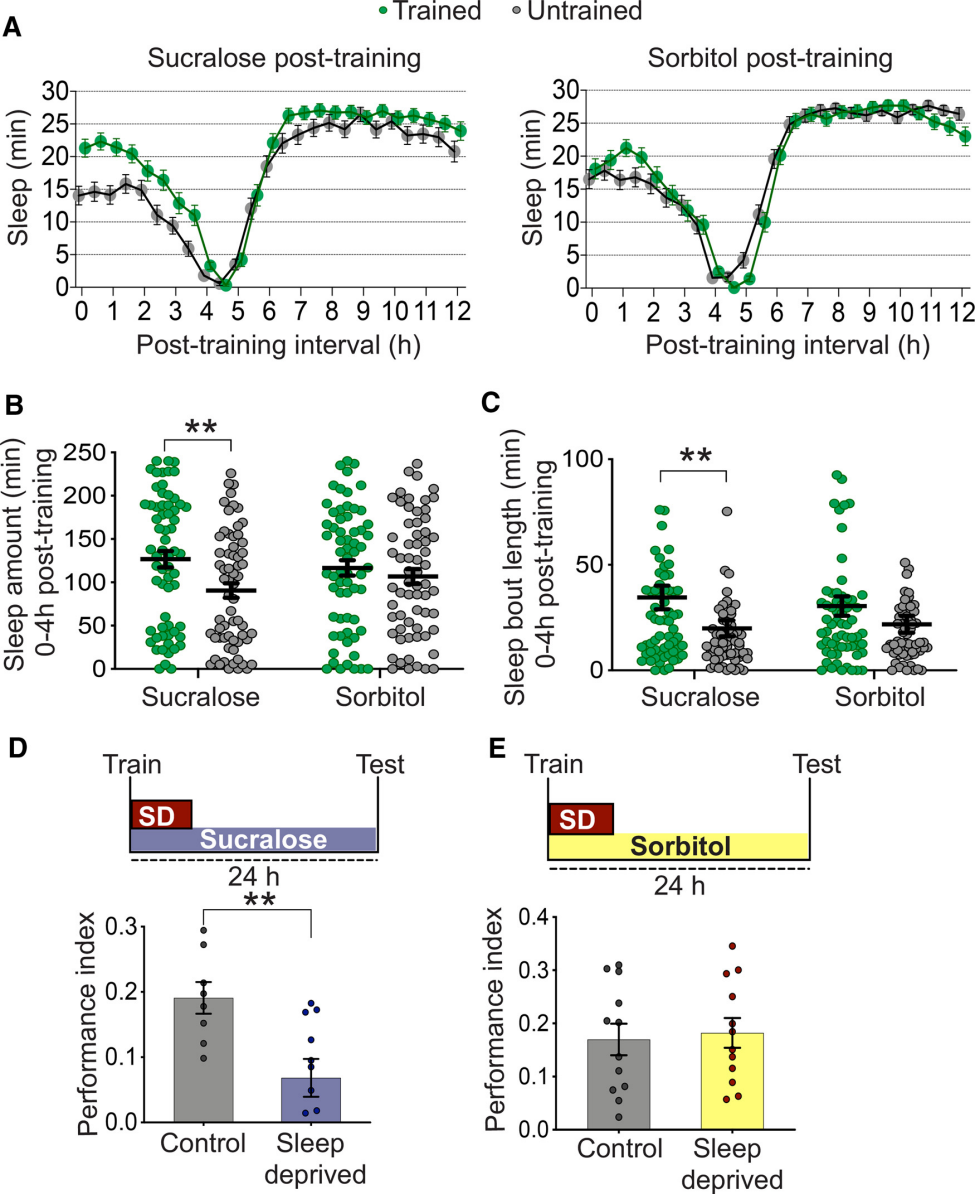
Sweet taste, but not nutritive value, induces the need for sleep in memory consolidation. ***A***, Flies trained at ZT6 and then kept on sucralose sleep better than untrained control flies. In contrast, trained and untrained flies show comparable sleep when kept on sorbitol following training. Total sleep in the first 4 h after training (ZT8-ZT12) is quantified in ***B*** (*n* = 64). ***C***, Demonstrating improved sleep consolidation, trained flies on sucralose demonstrate higher sleep bout length than untrained flies, while flies on sorbitol show comparable sleep bout length between trained and untrained groups (*n* = 64). ***D***, Exposure to 6 h of sleep deprivation affects long-term memory in flies kept on sucralose after training (*n* ≥ 8). ***E***, Appetitive 24 h memory remained intact in flies sleep deprived while on sorbitol following training (*n* = 12). Data are mean ± SEM. **p < 0.01: multiple two-sided *t* tests followed by Bonferroni correction (***B***), Mann–Whitney *U* tests (***C***), and two-sided *t* tests (***D***).

Sleep is necessary for the consolidation of memory in flies kept on arabinose following training (Chouhan et al., 2021). As arabinose is a sweet but nonmetabolizable sugar, these data suggested that sweet taste is sufficient to trigger sleep-dependent memory consolidation (Wigglesworth, 1949). To further assess the role of sweet taste in sleep-dependent memory, we tested sleep and memory in flies kept on either sucralose, a sweet but non-nutritive sugar, or sorbitol, a tasteless but nutritive substrate. Groups of starved flies were first trained at ZT6 to associate a sucrose reward with an odor, and then sleep was assayed in individual flies introduced into locomotor tubes with either sucralose or sorbitol. Untrained flies were presented with sucrose without an odor. Consistent with previous work with arabinose, trained flies on sucralose slept better than untrained controls (Fig. 1*A*). In the first 4 h after training (ZT8 to ZT12), trained flies on sucralose show higher sleep quantity and better quality, manifest as longer sleep bouts, than untrained flies (Fig. 1*B*, two-sided *t* tests followed by Bonferroni correction, *p* (trained vs untrained) = 0.009417; Fig. 1*C*, Mann–Whitney *U* tests, *p* (trained vs untrained) = 0.0052; Table 1). In contrast, flies kept on sorbitol after training showed comparable sleep between trained and untrained groups (Fig. 1*A–C*).

**Table 1.** Statistical analysis*^a^*

Figure	Distribution	Statistical analysis	Comparison groups (n)	Results
1 *B*	Normal	Multiple 2-sided *t* tests followed by Bonferroni correction	Sucralose: trained (64) vs untrained (64)	*t*_(126)_ = 2.878; *p* = 0.009417**
			Sorbitol: trained (64) vs untrained (64)	*t*_(126)_ = 0.7993; *p* = 0.851195
1 *C*	Non-normal	Mann–Whitney *U* tests	Sucralose: trained (64) vs untrained (64)	*p* = 0.0052**
			Sorbitol: trained (64) vs untrained (64)	*p* = 0.0529
1 *D*	Normal	2-sided *t* tests	Control (8) vs sleep-deprived (11)	*t*_(17)_ = 3.078; *p* = 0.0068**
1 *E*	Normal	2-sided *t* tests	Control (12) vs sleep-deprived (12)	*t*_(22)_ = 0.3032; *p* = 0.7646
2 *A*	Sleep amount: normal	Multiple 2-sided *t* tests followed by Bonferroni correction	UAS-*shi^ts1^*/^+^: trained (32) vs untrained (32)	*t*_(62)_ = 4.272; *p* = 0.000203***
			Gr64f-Gal4/^+^: trained (32) vs untrained (32)	*t*_(62)_ = 3.158; *p* = 0.007366**
			UAS-*shi^ts1^*/Gr64f-Gal4: trained (32) vs untrained (30)	*t*_(59)_ = 2.578; *p* = 0.037386*
	Sleep bout length: non-normal	Mann–Whitney *U* tests	UAS-*shi^ts1^*/^+^: trained (32) vs untrained (32)	*p* = 0.0341*
			Gr64f-Gal4/^+^: trained (32) vs untrained (32)	*p* = 0.0438*
			UAS-*shi^ts1^*/Gr64f-Gal4: trained (32) vs untrained (30)	*p* = 0.0036**
2 *B*	Sleep amount: normal	Multiple 2-sided *t* tests followed by Bonferroni correction	UAS-*shi^ts1^*/^+^: trained (32) vs untrained (32)	*t*_(62)_ = 3.003; *p* = 0.011545*
			Gr64f-Gal4/^+^: trained (62) vs untrained (63)	*t*_(121)_ = 2.579; *p* = 0.033312*
			UAS-*shi^ts1^*/Gr64f-Gal4: trained (32) vs untrained (32)	*t*_(62)_ = 0.2024; *p* > 0.999999
	Sleep bout length: non-normal	Mann–Whitney *U* tests	UAS-*shi^ts1^*/^+^: trained (32) vs untrained (32)	*p* = 0.0207*
			Gr64f-Gal4/^+^: trained (62) vs untrained (63)	*p* = 0.0112*
			UAS-*shi^ts1^*/Gr64f-Gal4: trained (32) vs untrained (32)	*p* = 0.2656
2 *C*	Normal	One-way ANOVA followed by Tukey's *post hoc* test		*F*_(2,15)_ = 0.08762; *p* = 0.9166
			UAS-*shi^ts1^*/^+^ (6) vs Gr64f-Gal4/^+^ (6)	*p* = 0.9921
			UAS-*shi^ts1^*/^+^ (6) vs UAS-*shi^ts1^*/Gr64f-Gal4 (6)	*p*= 0.9556
			Gr64f-Gal4/^+^ (6) vs UAS-*shi^ts1^*/Gr64f-Gal4 (6)	*p* = 0.9132
2 *D*	Normal	One-way ANOVA followed by Tukey's *post hoc* test		*F*_(2,21)_ = 13.95; *p* = 0.0001
			UAS-*shi^ts1^*/^+^ (8) vs Gr64f-Gal4/^+^ (10)	*p* = 0.2554
			UAS-*shi^ts1^*/^+^ (8) vs UAS-*shi^ts1^*/Gr64f-Gal4 (6)	*p* = 0.0001***
			Gr64f-Gal4/^+^ (10) vs UAS-*shi^ts1^*/Gr64f-Gal4 (6)	*p* = 0.0022**
2 *E*	Normal	One-way ANOVA followed by Tukey's *post hoc* test		*F*_(2,17)_ = 0.7574; *p* = 0.4841
			UAS-*shi^ts1^*/^+^ (6) vs Gr64f-Gal4/^+^ (6)	*p*= 0.6877
			UAS-*shi^ts1^*/^+^ (6) vs UAS-*shi^ts1^*/Gr64f-Gal4 (8)	*p* = 0.9455
			Gr64f-Gal4/^+^ (6) vs UAS-*shi^ts1^*/Gr64f-Gal4 (8)	*p* = 0.4631
3 *A*	Normal	One-way ANOVA followed by Tukey's *post hoc* test		*F*_(2,21)_ = 1.091; *p* = 0.3540
			UAS-*TrpA1*/^+^ (8) vs Gr64f-Gal4/^+^ (6)	*p* = 0.3842
			UAS-*TrpA1*/^+^ (8) vs UAS-*TrpA1*/Gr64f-Gal4 (10)	*p* = 0.99
			Gr64f-Gal4/^+^ (6) vs UAS-*TrpA1*/Gr64f-Gal4 (10)	*p* = 0.4174
3 *B*	Normal	One-way ANOVA followed by Tukey's *post hoc* test		*F*_(2,17)_ = 5.754; *p* = 0.0123
			UAS-*TrpA1*/^+^ (8) vs Gr64f-Gal4/^+^ (6)	*p* = 0.9547
			UAS-*TrpA1*/^+^ (8) vs UAS-*TrpA1*/Gr64f-Gal4 (6)	*p* = 0.0144*
			Gr64f-Gal4/^+^ (6) vs UAS-*TrpA1*/Gr64f-Gal4 (6)	*p* = 0.0382*
3 *C*	Normal	One-way ANOVA followed by Tukey's *post hoc* test		*F*_(2,17)_ = 0.7676; *p* = 0.4796
			UAS-*TrpA1*/^+^ (8) vs Gr64f-Gal4/^+^ (6)	*p* = 0.4507
			UAS-*TrpA1*/^+^ (8) vs UAS-*TrpA1*/Gr64f-Gal4 (6)	*p* = 0.8009
			Gr64f-Gal4/^+^ (6) vs UAS-*TrpA1*/Gr64f-Gal4 (6)	*p* = 0.8451
4 *A*	Restrictive: normal	One-way ANOVA followed by Tukey's *post hoc* test		*F*_(2,21)_ = 5.730; *p* = 0.0103
			UAS-*shi^ts1^*/^+^ (8) vs R58E02/^+^ (6)	*p* = 0.9773
			UAS-*shi^ts1^*/^+^ (8) vs UAS-*shi^ts1^*/R58E02 (10)	*p* = 0.0161*
			R58E02/^+^ (6) vs UAS-*shi^ts1^*/R58E02 (10)	*p* = 0.0436*
	Permissive: normal	One-way ANOVA followed by Tukey's *post hoc* test		*F*_(2,21)_ = 0.8667; *p* = 0.4381
			UAS-*shi^ts1^*/^+^ (6) vs R58E02/^+^ (6)	*p* = 0.4474
			UAS-*shi^ts1^*/^+^ (6) vs UAS-*shi^ts1^*/R58E02 (8)	*p* = 0.5636
			R58E02/^+^ (6) vs UAS-*shi^ts1^*/R58E02 (8)	*p* = 0.956
4 *B*	Restrictive: normal	One-way ANOVA followed by Tukey's *post hoc* test		*F*_(6,46)_ = 5.960; *p* = 0.0001
			UAS-*shi^ts1^*/^+^ (6) vs MB299B/^+^ (9)	*p* > 0.9999
			UAS-*shi^ts1^*/^+^ (6) vs UAS-*shi^ts1^*/MB299B (8)	*p* = 0.0452*
			UAS-*shi^ts1^*/^+^ (6) vs MB196B/^+^ (6)	*p* = 0.9992
			UAS-*shi^ts1^*/^+^ (6) vs UAS-*shi^ts1^*/MB196B (6)	*p* > 0.9999
			UAS-*shi^ts1^*/^+^ (6) vs MB056B/^+^ (9)	*p* = 0.5063
			UAS-*shi^ts1^*/^+^ (6) vs UAS-*shi^ts1^*/MB056B (9)	*p* = 0.865
			MB299B/^+^ (9) vs UAS-*shi^ts1^*/MB299B (8)	*p* = 0.0099**
			MB299B/^+^ (9) vs MB196B/^+^ (6)	*p* > 0.9999
			MB299B/^+^ (9) vs UAS-*shi^ts1^*/MB196B (6)	*p* > 0.9999
			MB299B/^+^ (9) vs MB056B/^+^ (9)	*p* = 0.5097
			MB299B/^+^ (9) vs UAS-*shi^ts1^*/MB056B (9)	*p* = 0.8962
			UAS-*shi^ts1^*/MB299B (8) vs MB196B/^+^ (6)	*p* = 0.0121*
			UAS-*shi^ts1^*/MB299B (8) vs UAS-*shi^ts1^*/MB196B (6)	*p* = 0.0214*
			UAS-*shi^ts1^*/MB299B (8) vs MB056B/^+^ (9)	*p* < 0.0001***
			UAS-*shi^ts1^*/MB299B (8) vs UAS-*shi^ts1^*/MB056B (9)	*p* = 0.0003***
			MB196B/^+^ (6) vs UAS-*shi^ts1^*/MB196B (6)	*p* > 0.9999
			MB196B/^+^ (6) vs MB056B/^+^ (9)	*p* = 0.8154
			MB196B/^+^ (6) vs UAS-*shi^ts1^*/MB056B (9)	*p* = 0.988
			UAS-*shi^ts1^*/MB196B (6) vs MB056B/^+^ (9)	*p* = 0.6955
			UAS-*shi^ts1^*/MB056B (9) vs UAS-*shi^ts1^*/MB196B (6)	*p* = 0.9586
			MB056B/^+^ (9) vs UAS-*shi^ts1^*/MB056B (9)	*p* = 0.9928
	Permissive: normal	One-way ANOVA followed by Tukey's *post hoc* test		*F*_(6,46)_ = 5.960; *p* = 0.0001
			UAS-*shi^ts1^*/^+^ (7) vs MB299B/^+^ (6)	*p* = 0.275
			UAS-*shi^ts1^*/^+^ (7) vs UAS-*shi^ts1^*/MB299B (6)	*p* = 0.9988
			UAS-*shi^ts1^*/^+^ (7) vs MB196B/^+^ (6)	*p* > 0.9999
			UAS-*shi^ts1^*/^+^ (7) vs UAS-*shi^ts1^*/MB196B (6)	*p* = 0.9988
			UAS-*shi^ts1^*/^+^ (7) vs MB056B/^+^ (7)	*p* = 0.9991
			UAS-*shi^ts1^*/^+^ (7) vs UAS-*shi^ts1^*/MB056B (6)	*p* = 0.9206
			MB299B/^+^ (6) vs UAS-*shi^ts1^*/MB299B (6)	*p* = 0.5982
			MB299B/^+^ (6) vs MB196B/^+^ (6)	*p* = 0.3478
			MB299B/^+^ (6) vs UAS-*shi^ts1^*/MB196B (6)	*p* = 0.5976
			MB299B/^+^ (6) vs MB056B/^+^ (7)	*p* = 0.5291
			MB299B/^+^ (6) vs UAS-*shi^ts1^*/MB056B (6)	*p* = 0.9122
			UAS-*shi^ts1^*/MB299B (6) vs MB196B/^+^ (6)	*p* = 0.9996
			UAS-*shi^ts1^*/MB299B (6) vs UAS-*shi^ts1^*/MB196B (6)	*p* > 0.9999
			UAS-*shi^ts1^*/MB299B (6) vs MB056B/^+^ (7)	*p* > 0.9999
			UAS-*shi^ts1^*/MB299B (6) vs UAS-*shi^ts1^*/MB056B (6)	*p* = 0.9965
			MB196B/^+^ (6) vs UAS-*shi^ts1^*/MB196B (6)	*p* = 0.9996
			MB196B/^+^ (6) vs MB056B/^+^ (7)	*p* = 0.9997
			MB196B/^+^ (6) vs UAS-*shi^ts1^*/MB056B (6)	*p* = 0.9485
			UAS-*shi^ts1^*/MB196B (6) vs MB056B/^+^ (7)	*p* > 0.9999
			UAS-*shi^ts1^*/MB056B (6) vs UAS-*shi^ts1^*/MB196B (6)	*p* = 0.9965
			MB056B/^+^ (7) vs UAS-*shi^ts1^*/MB056B (6)	*p* = 0.9939
4 *C*	Restrictive: normal	One-way ANOVA followed by Tukey's *post hoc* test		*F*_(6,42)_ = 4.996; *p* = 0.0006
			UAS-*shi^ts1^*/^+^ (6) vs MB299B/^+^ (8)	*p* = 0.7519
			UAS-*shi^ts1^*/^+^ (6) vs UAS-*shi^ts1^*/MB299B (7)	*p* = 0.5535
			UAS-*shi^ts1^*/^+^ (6) vs MB196B/^+^ (6)	*p* = 0.6901
			UAS-*shi^ts1^*/^+^ (6) vs UAS-*shi^ts1^*/MB196B (6)	*p* = 0.9075
			UAS-*shi^ts1^*/^+^ (6) vs MB056B/^+^ (8)	*p* = 0.9829
			UAS-*shi^ts1^*/^+^ (6) vs UAS-*shi^ts1^*/MB056B (8)	*p* = 0.0059**
			MB299B/^+^ (8) vs UAS-*shi^ts1^*/MB299B (7)	*p* = 0.9997
			MB299B/^+^ (8) vs MB196B/^+^ (6)	*p* > 0.9999
			MB299B/^+^ (8) vs UAS-*shi^ts1^*/MB196B (6)	*p* > 0.9999
			MB299B/^+^ (8) vs MB056B/^+^ (8)	*p* = 0.194
			MB299B/^+^ (8) vs UAS-*shi^ts1^*/MB056B (8)	*p* = 0.1508
			UAS-*shi^ts1^*/MB299B (7) vs MB196B/^+^ (6)	*p* > 0.9999
			UAS-*shi^ts1^*/MB299B (7) vs UAS-*shi^ts1^*/MB196B (6)	*p* = 0.9962
			UAS-*shi^ts1^*/MB299B (7) vs MB056B/^+^ (8)	*p* = 0.1036
			UAS-*shi^ts1^*/MB299B (7) vs UAS-*shi^ts1^*/MB056B (8)	*p* = 0.3563
			MB196B/^+^ (6) vs UAS-*shi^ts1^*/MB196B (6)	*p* = 0.9994
			MB196B/^+^ (6) vs MB056B/^+^ (8)	*p* = 0.1832
			MB196B/^+^ (6) vs UAS-*shi^ts1^*/MB056B (8)	*p* = 0.3185
			UAS-*shi^ts1^*/MB196B (6) vs MB056B/^+^ (8)	*p* = 0.406
			UAS-*shi^ts1^*/MB056B (8) vs UAS-*shi^ts1^*/MB196B (6)	*p* = 0.1333
			MB056B/^+^ (8) vs UAS-*shi^ts1^*/MB056B (8)	*p* = 0.0002***
	Permissive: normal	One-way ANOVA followed by Tukey's *post hoc* test		*F*_(6,42)_ = 0.6352; *p* = 0.7013
			UAS-*shi^ts1^*/^+^ (6) vs MB299B/^+^ (6)	*p* > 0.9999
			UAS-*shi^ts1^*/^+^ (6) vs UAS-*shi^ts1^*/MB299B (10)	*p* = 0.9865
			UAS-*shi^ts1^*/^+^ (6) vs MB196B/^+^ (6)	*p* = 0.8539
			UAS-*shi^ts1^*/^+^ (6) vs UAS-*shi^ts1^*/MB196B (6)	*p* = 0.9343
			UAS-*shi^ts1^*/^+^ (6) vs MB056B/^+^ (8)	*p* = 0.9997
			UAS-*shi^ts1^*/^+^ (6) vs UAS-*shi^ts1^*/MB056B (7)	*p* = 0.9996
			MB299B/^+^ (6) vs UAS-*shi^ts1^*/MB299B (10)	*p* = 0.9267
			MB299B/^+^ (6) vs MB196B/^+^ (6)	*p* = 0.6984
			MB299B/^+^ (6) vs UAS-*shi^ts1^*/MB196B (6)	*p* = 0.8221
			MB299B/^+^ (6) vs MB056B/^+^ (8)	*p* = 0.9918
			MB299B/^+^ (6) vs UAS-*shi^ts1^*/MB056B (7)	*p* = 0.9912
			UAS-*shi^ts1^*/MB299B (10) vs MB196B/^+^ (6)	*p* = 0.9937
			UAS-*shi^ts1^*/MB299B (10) vs UAS-*shi^ts1^*/MB196B (6)	*p* = 0.9995
			UAS-*shi^ts1^*/MB299B (10) vs MB056B/^+^ (8)	*p* = 0.9997
			UAS-*shi^ts1^*/MB299B (10) vs UAS-*shi^ts1^*/MB056B (7)	*p* = 0.9999
			MB196B/^+^ (6) vs UAS-*shi^ts1^*/MB196B (6)	*p* > 0.9999
			MB196B/^+^ (6) vs MB056B/^+^ (8)	*p* = 0.9552
			MB196B/^+^ (6) vs UAS-*shi^ts1^*/MB056B (7)	*p* = 0.9666
			UAS-*shi^ts1^*/MB196B (6) vs MB056B/^+^ (8)	*p* = 0.9887
			UAS-*shi^ts1^*/MB056B (7) vs UAS-*shi^ts1^*/MB196B (6)	*p* = 0.9922
			MB056B/^+^ (8) vs UAS-*shi^ts1^*/MB056B (7)	*p* > 0.9999
5 *A*	Non-normal	Mann–Whitney *U* tests	Trained: fed (17) vs starved (13)	*p* = 0.0005***
			Untrained: fed (10) vs starved (7)	*p* = 0.0012**
			Fed: trained (17) vs untrained (10)	*p* = 0.0588
			Starved: trained (13) vs untrained (7)	*p* = 0.8773
5 *B*	Non-normal	Mann–Whitney *U* tests	Trained: fed (7) vs starved (14)	*p* < 0.0001***
			Untrained: fed (9) vs starved (11)	*p* = 0.2299
			Fed: trained (7) vs untrained (9)	*p* = 0.6806
			Starved: trained (14) vs untrained (11)	*p* < 0.0001***
6 *A*	Non-normal	Mann–Whitney *U* tests	Arabinose (7) vs sorbitol (12)	*p* < 0.0001***
			Sucralose (10) vs sorbitol (12)	*p* = 0.0001***
			Arabinose (7) vs sucralose (10)	*p* = 0.1331
6 *B*	Non-normal	Mann–Whitney *U* tests	Arabinose (8) vs sorbitol (10)	*p* = 0.0003***
			Sucralose (9) vs sorbitol (10)	*p* < 0.0001***
			Arabinose (8) vs sucralose (9)	*p* = 0.1672

*^a^*A summary of statistical analyses shown in this study, which includes information on statistical tests, data distribution, and *p* values.

We next tested whether disrupting sleep after training affects long-term memory. Groups of trained flies were kept on either sucralose or sorbitol and sleep-deprived for the first 6 h after training, and then tested for memory after 24 h. We found that flies on sucralose require sleep to form memory as sleep deprivation affected memory performance (Fig. 1*D*, two-sided *t* test, *p* (control vs sleep-deprived) = 0.0068; Table 1). In contrast, sleep was dispensable for memory consolidation in flies kept on sorbitol (Fig. 1*E*). Therefore, flies kept on sucralose form sleep-dependent memory, while flies on sorbitol form sleep-independent memory in an appetitive conditioning paradigm. Together, these results suggest that a sweet stimulus is essential for sleep-dependent memory consolidation.

### Sweet-sensing neurons mediate sleep-dependent memory consolidation

**Figure 2.**
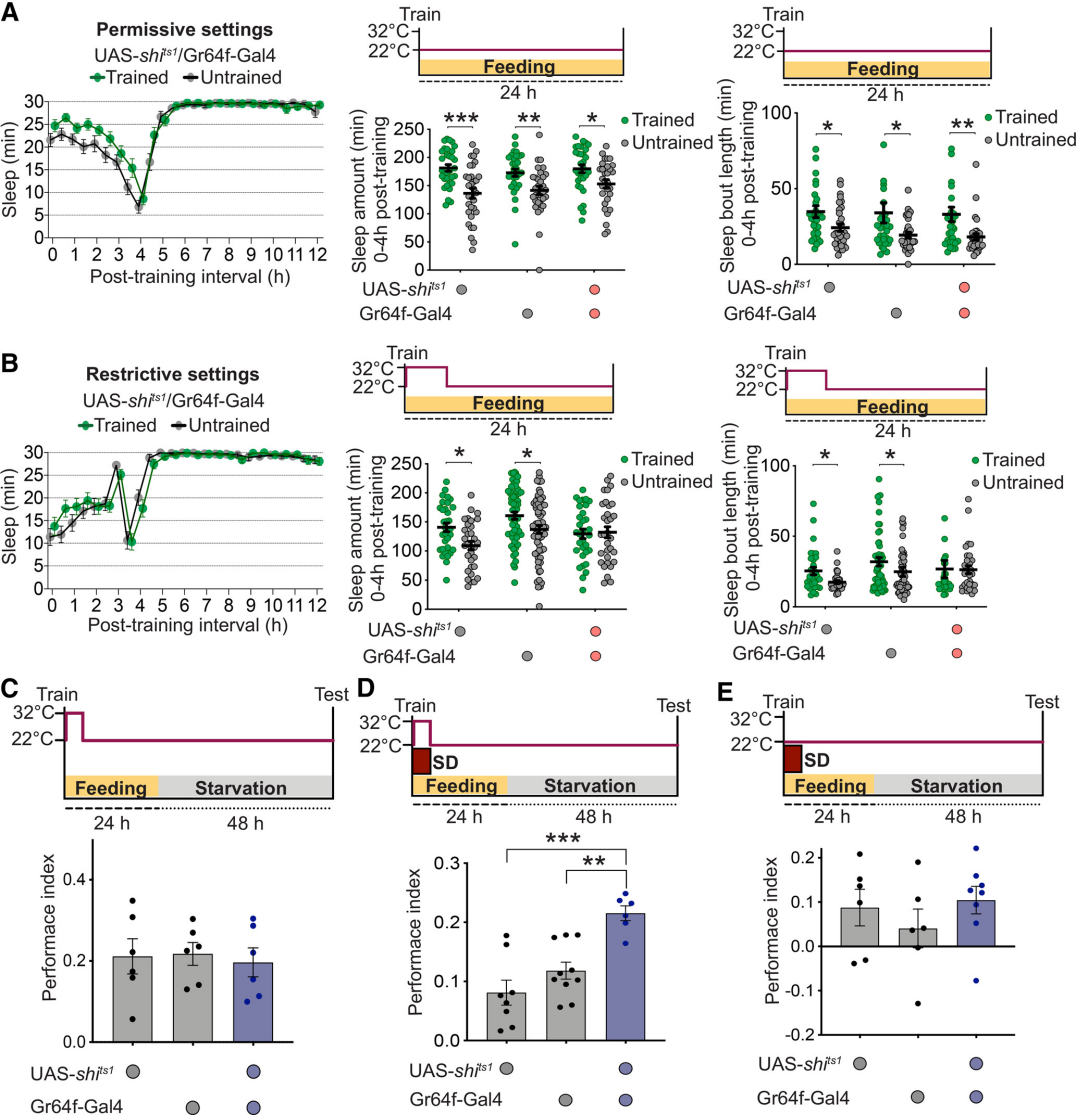
Activity in Gr64f^+^ neurons is essential for sleep-dependent memory. ***A***, Experimental (flies expressing *shibire* in Gr64f neurons) and genetic control flies demonstrate a robust increase in post-training sleep when fed at permissive settings. Sleep amount and sleep bout length in the ZT8-ZT12 interval are depicted (*n* = 32 for each genotype). ***B***, Fed UAS-*shibire^ts1^*/Gr64f-Gal4 flies show comparable sleep between trained and untrained flies; however, trained UAS- and Gal4- control flies demonstrate a robust post-training sleep enhancement at restrictive settings. Total sleep and sleep bout length in the first 4 h after training are depicted (*n* = 32 per genotype). ***C***, Silencing Gr64f^+^ neurons for 6 h after training has no impact on long-term memory in fed flies (*n* = 6). ***D***, Six hours of sleep deprivation following training at 32°C affects long-term memory in genetic controls; however, UAS-*shibire^ts1^*/Gr64f-Gal4 flies demonstrate a robust and significantly better memory performance in fed settings (*n* ≥ 6). ***E***, At permissive temperature of 22°C, sleep disruption affects memory consolidation in all genotypes as experimental and genetic controls demonstrate lower but comparable memory scores (*n* ≥ 6). Data are mean ± SEM. *p < 0.05; **p < 0.01; ***p < 0.001: multiple two-sided *t* tests followed by Bonferroni correction (***A***,***B***: Total sleep amount), Mann–Whitney *U* tests (***A***,***B***: Sleep bout length), and one-way ANOVA followed by Tukey *post hoc* tests (***D***).

Sweet taste perception in flies is mediated by gustatory receptors on the proboscis and tarsi. Based on findings that the Gr64f receptor is broadly expressed in sweet-sensing neurons (Jiao et al., 2008), we asked whether Gr64f^+^ neurons are required for the consolidation of sleep-dependent memory. We used the UAS-Gal4 system to express a dominant negative and temperature-sensitive allele of dynamin called *shibire^ts1^* in Gr64f^+^ neurons and first assayed a role for these neurons in the sleep increase seen after appetitive training. *shibire^ts1^* blocks synaptic transmission when flies are placed at the restrictive temperature (32°C) but is ineffective at 22°C, the permissive temperature (Kitamoto, 2001). Flies were first trained in groups to form an odor-reward association at permissive settings, and then individual flies were placed in locomotor tubes with standard cornmeal fly food for sleep assessment at either 22°C or 32°C. Control and experimental trained flies showed a significant increase in sleep after training when kept at 22°C (Fig. 2*A*, Sleep quantity: two-sided *t* tests followed by Bonferroni correction, *p* (UAS-*shibire^ts1^*/^+^: trained vs untrained) = 0.000203, *p* (Gr64f-Gal4/^+^: trained vs untrained) = 0.007366, *p* (UAS-*shibire^ts1^*/Gr64f-Gal4: trained vs untrained) = 0.037386; Sleep bout length: Mann–Whitney *U* tests, *p* (UAS-*shibire^ts1^*/^+^: trained vs untrained) = 0.0341, *p* (Gr64f-Gal4/^+^: trained vs untrained) = 0.0438, *p* (UAS-*shibire^ts1^*/Gr64f-Gal4: trained vs untrained) = 0.0036; Table 1). However, silencing Gr64f^+^ neurons affected sleep increase after training as trained experimental flies showed no change in sleep quality and quantity compared with untrained flies, although a training-dependent increase was observed in genetic control flies (Fig. 2*B*, Sleep quantity: two-sided *t* tests followed by Bonferroni correction, *p* (UAS-*shibire^ts1^*/^+^: trained vs untrained) = 0.011545, *p* (Gr64f-Gal4/^+^: trained vs untrained) = 0.033312, *p* (UAS-*shibire^ts1^*/Gr64f-Gal4: trained vs untrained) > 0.999999; Sleep bout length: Mann–Whitney *U* tests, *p* (UAS-*shibire^ts1^*/^+^: trained vs untrained) = 0.0207, *p* (Gr64f-Gal4/^+^: trained vs untrained) = 0.0112, *p* (UAS-*shibire^ts1^*/Gr64f-Gal4: trained vs untrained) = 0.2656; Table 1).

We next assessed whether silencing Gr64f^+^ neurons affects the requirement for sleep in memory consolidation. Trained flies were kept in cornmeal food vials and placed at 32°C for the first 6 h following training, and then maintained at permissive settings. Memory was assayed following a 48 h restarvation period, and was per se not affected by silencing of the Gr64f^+^ neurons (Fig. 2*C*). However, silencing Gr64f^+^ neurons resulted in the consolidation of sleep-independent memory in fed flies; while controls showed a decrement in memory with sleep deprivation, no such decrease occurred in flies expressing *shibire^ts1^* in Gr64f^+^ neurons at restrictive temperature (Fig. 2*D*, one-way ANOVA followed by Tukey's *post hoc* test, *F*_(2,21)_ = 13.95, *p* = 0.0001; *p* (UAS-*shibire^ts1^*/^+^ vs UAS-*shibire^ts1^*/Gr64f-Gal4) = 0.0001, *p* (Gr64f-Gal4/^+^ vs UAS-*shibire^ts1^*/Gr64f-Gal4) = 0.0022; Fig. 2*E*; Table 1).

**Figure 3.**
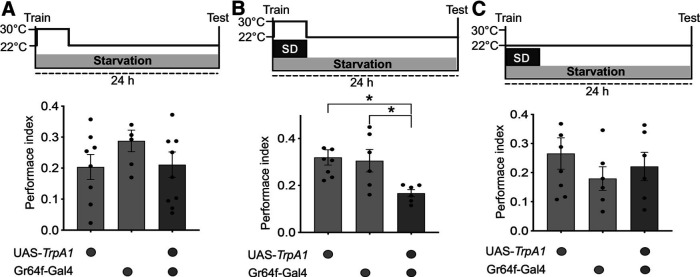
Stimulation of Gr64f^+^ neurons is sufficient to drive sleep-dependent memory. ***A***, Thermogenetic activation of Gr64f^+^ neurons for 6 h after training does not affect memory consolidation in starved flies (*n* ≥ 6). ***B***, At 30°C, 6 h of sleep deprivation after training under starvation conditions has no impact on memory in UAS- and Gal4- control flies, but affects long-term memory in UAS-*TrpA1*/Gr64f-Gal4 flies (*n* ≥ 6). ***C***, At permissive settings, experimental and genetic control flies show robust and comparable memory scores when exposed to 6 h of sleep deprivation following training (*n* ≥ 6). Data are mean ± SEM. *p < 0.05 (one-way ANOVA followed by Tukey *post hoc* tests).

As noted above, starved flies form sleep-independent memory (Chouhan et al., 2021). Given that sleep and memory are coupled by sweet taste, which is sensed by Gr64f^+^ neurons, we asked whether stimulating these neurons in starved flies would induce a switch to sleep-dependent memory consolidation. To test this, we used a temperature-gated depolarizing cation channel, TrpA1, that activates neurotransmission at 30°C but remains ineffective at 22°C. Transient activation of Gr64f^+^ neurons with TrpA1 did not affect memory consolidation in unperturbed starved flies, but it made the memory consolidation dependent on sleep (Fig. 3*A*). Exposure to 6 h of sleep deprivation following training impacted long-term memory in experimental flies but not in genetic control flies at restrictive settings (Fig. 3*B*, one-way ANOVA followed by Tukey's *post hoc* test, *F*_(2,17)_ = 5.754, *p* = 0.0123; *p* (UAS-*TrpA1*/^+^ vs UAS-*TrpA1*/Gr64f-Gal4) = 0.0144, *p* (Gr64f-Gal4/^+^ vs UAS- *TrpA1*/Gr64f-Gal4) = 0.0382; Table 1). At the permissive temperature of 22°C, sleep deprivation had no effect on memory scores of experimental or genetic control flies (Fig. 3*C*). Together, these findings confirm that sweet taste information transduced through Gr64f^+^ neurons drives the consolidation of sleep-dependent memory in an appetitive conditioning paradigm.

### Distinct PAM DANs mediate memory consolidation in fed and starved flies

**Figure 4.**
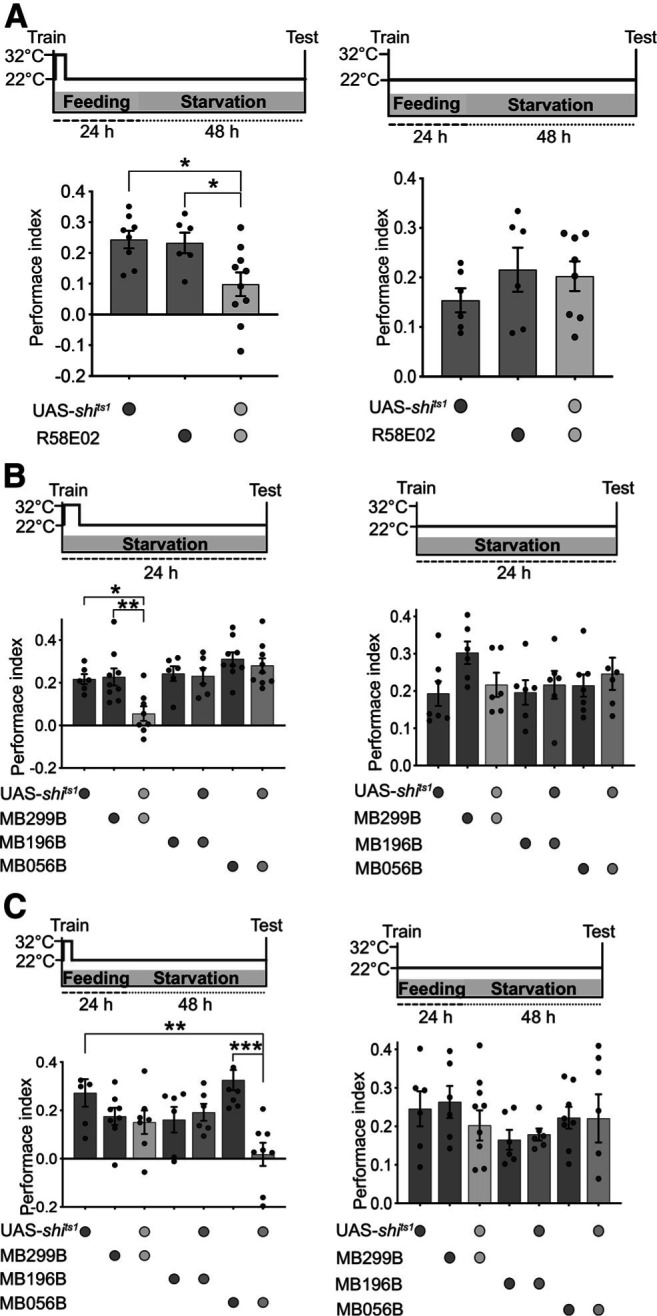
Discrete PAM DANs are recruited for memory consolidation in fed versus starved flies. ***A***, Silencing PAM DANs for 4 h following training affects memory consolidation in fed flies (*n* ≥ 6). *shibire^ts^* does not affect memory in flies maintained on food at 22°C (*n* ≥ 6). ***B***, Neurotransmission from PAM-α1 is essential for memory consolidation in starved flies (*n* ≥ 6). Long-term memory is comparable between experimental and genetic controls at permissive settings (*n* ≥ 6). ***C***, Blocking the activity of PAM-β′2mp for 4 h after training affects memory consolidation in fed flies (*n* = 8). *shibire^ts^* has no effect on memory in flies maintained on food at permissive temperature (*n* ≥ 6). Data are mean ± SEM. *p < 0.05; **p < 0.01; ***p < 0.001; one-way ANOVA followed by Tukey *post hoc* tests.

Most previous studies of appetitive memory starved flies after conditioning, so little is known about the circuitry in fed flies. However, we showed that the circuit for sleep-dependent memory in fed flies is different at the level of the MBs and the PPL1 dopamine cluster (Chouhan et al., 2021). Yet another group of memory-relevant dopamine neurons is the PAM cluster, which provides positive valence during olfactory conditioning and is required for long-term memory consolidation in starved flies (Ichinose et al., 2015; Yamagata et al., 2015). We first tested whether PAM DANs as a whole are required for memory consolidation in fed flies. Following appetitive training, flies expressing *shibire^ts1^* in PAM DANs showed impaired long-term memory performance compared with UAS- and GAL4- control flies at the restrictive temperature, but comparable memory scores at the permissive temperature (Fig. 4*A*, one-way ANOVA followed by Tukey's *post hoc* test, *F*_(2,21)_ = 5.73, *p* = 0.0103; *p* (UAS-*shibire^ts1^*/^+^ vs UAS-*shibire^ts1^*/R58E02) = 0.0161, *p* (R58E02/^+^ vs UAS-*shibire^ts1^*/R58E02) = 0.0436; Table 1). This indicates that dopaminergic neurons in the PAM cluster are required for memory consolidation in both fed and starved flies.

We next used split-Gal4 lines to functionally restrict expression of *shibire* to specific subsets of neurons in the PAM cluster (Aso et al., 2014). Consistent with a previous study (Ichinose et al., 2015), silencing PAM-α1 DANs, with MB299B split-Gal4 line, affected long-term memory in starved flies (Fig. 4*B*, one-way ANOVA followed by Tukey's *post hoc* test, *F*_(6,46)_ = 5.96, *p* = 0.0001; *p* (UAS-*shibire^ts1^*/^+^ vs UAS-*shibire^ts1^*/MB299B) = 0.0452, *p* (MB299B/^+^ vs UAS-*shibire^ts1^*/MB299B) = 0.0099; Table 1). Surprisingly, if UAS-*shibire^ts1^*/MB299B flies were moved to a food vial at 32°C for 4 h after training, then appetitive long-term memory was comparable to that of genetic controls, indicating that these neurons are not relevant for memory consolidation in fed flies (Fig. 4*C*).

Individual DANs synapse onto a single, or at most two, MB compartments that are innervated by a corresponding MBON (Aso et al., 2014). Since γ2α′1 MBONs, which are involved in sleep-dependent memory consolidation, connect to specific PAM DANs (Aso et al., 2014; Chouhan et al., 2021), we next tested whether signaling from these PAM DANs is required for memory consolidation in fed flies. We first used the MB196B split-Gal4 line that labels PAM-β′2a, PAM-γ3, PAM-γ4, PAM-γ4<γ1γ2, and PAM-γ5 DANs. Silencing PAM DANs labeled by MB196B for 4 h following training had no effect on memory performance in both fed and starved settings, indicating that these PAM DANs are dispensable for memory consolidation (Fig. 4*B*,*C*). PAM-β′2mp DANs signal reward during aversive conditioning for safety-memory acquisition and mediate feeding interactions, as do Gr64f^+^ neurons, in the Sip-Triggered Optogenetic Behavior Enclosure assay (Musso et al., 2019; Jacob and Waddell, 2020). Therefore, we next considered a role for PAM-β′2mp DANs, labeled with MB056B split-Gal4 line, in memory consolidation. At restrictive temperature, fed, but not starved, UAS-*shibire^ts1^*/MB056B flies demonstrated a significant decrease in long-term memory compared with UAS- and GAL4- control flies (Fig. 4*B*,*C*, one-way ANOVA followed by Tukey's *post hoc* test, *F*_(6,42)_ = 4.996, *p* = 0.0006; *p* (UAS-*shibire^ts1^*/^+^ vs UAS-*shibire^ts1^*/MB056B) = 0.0059, *p* (MB056B/^+^ vs UAS-*shibire^ts1^*/MB056B) = 0.0002; Table 1). Controls and experimental genotypes at permissive temperature showed similar long-term memory scores (Fig. 4*B*,*C*). These data demonstrate that PAM-α1 DANs are required for long-term memory in starved flies and PAM-β′2mp DANs are specifically needed for memory consolidation in fed flies.

### PAM-α1 and PAM-β′2mp respond differentially to training in a fed/starved state

**Figure 5.**
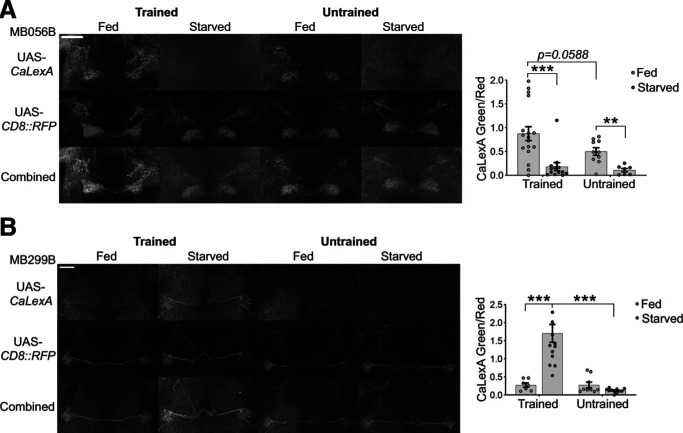
Distinct PAM DAN subsets are activated in response to food/starvation after training. Here, the Green Fluorescent Protein (GFP) signal reports aggregate changes in calcium while Red Fluorescent Protein (RFP) labels the cells. ***A***, GFP signal in PAM-β′2mp was substantially higher in fed flies compared with starved flies in both trained and untrained groups. Also, trained flies show a moderate, but insignificant, increase in calcium activity in PAM-β′2mp compared with untrained flies in fed settings (*n* ≥ 7). ***B***, Trained and starved flies demonstrate an increase in PAM-α1 activity compared with both fed flies and untrained controls (*n* ≥ 7). Data are mean ± SEM. **p < 0.01; ***p < 0.001; Mann–Whitney *U* tests.

We next investigated the functional heterogeneity in PAM DANs by using the CaLexA system to measure calcium changes in PAM-α1 and PAM-β′2mp DANs after training in fed and starved conditions. CaLexA reports changes in cellular calcium as a GFP signal that is transcriptionally activated by calcium-dependent nuclear import of the transcription factor fusion protein LexA-VP16-NFAT (Nuclear factor of activated T-cells) (Masuyama et al., 2012). Groups of flies were first trained to form an odor-reward association and placed in either a food vial or an agar vial for 2 h and then individual fly brains were prepared for imaging. The GFP signal in PAM-β′2mp DANs in fed flies was considerably higher than in starved flies in both trained and untrained groups (Fig. 5*A*, Mann–Whitney *U* tests, *p* (trained-fed vs trained-starved) = 0.0005, *p* (untrained-fed vs untrained-starved) = 0.0012; Table 1). Therefore, PAM-β′2mp DANs respond strongly to feeding; however, we also observed a moderate increase in their activity in trained compared with untrained flies in fed conditions (Fig. 5*A*, Mann–Whitney *U* tests, *p* (trained-fed vs untrained-fed) = 0.0588; Table 1). In contrast, we did not observe a feeding-dependent increase in the GFP signal in PAM-α1 DANs but found a substantial increase in their activity in trained and starved flies compared with trained and fed flies and untrained and starved flies, consistent with their role in memory consolidation in starved settings (Fig. 5*B*, Mann–Whitney *U* tests, *p* (trained-fed vs trained-starved) < 0.0001, *p* (trained-starved vs untrained-starved) < 0.0001; Table 1). These results indicate that feeding influences the selection of PAM DANs subsets for the consolidation of appetitive memory.

### Sweet taste drives the recruitment of PAM-β′2mp DANs for memory consolidation

**Figure 6.**
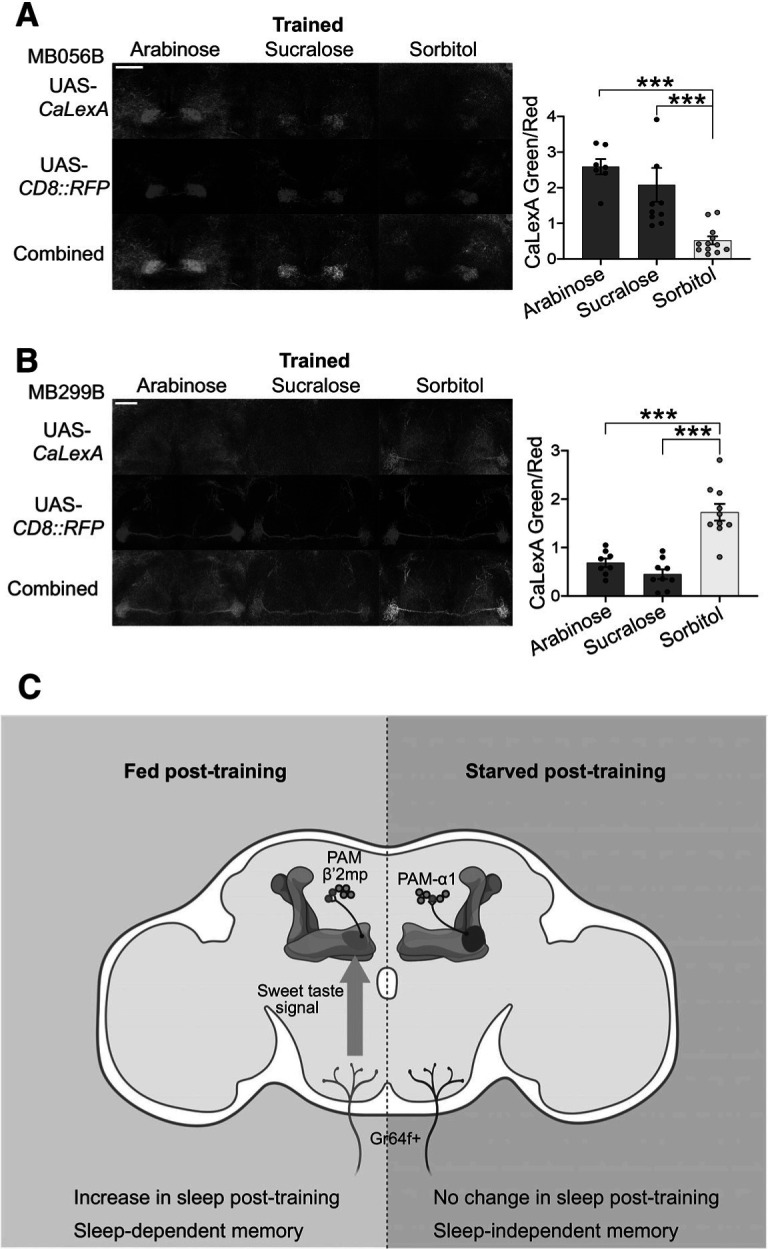
PAM DAN subsets respond differently to the presence of sweet taste after training. ***A***, Calcium response in PAM-β′2mp DANs is considerably higher in flies kept on arabinose or sucralose than in flies moved to sorbitol after training (*n* ≥ 7). ***B***, Trained flies show a higher increase in activity of PAM-α1 DANs when placed on sorbitol than on arabinose or sucralose following training (*n* ≥ 8). ***C***, Gr64f^+^ neurons mediated sweet taste signal activates PAM-b'2mp DANs in flies fed after training for sleep-dependent memory consolidation. In contrast, lack of sweet stimulus results in the recruitment of a distinct PAM DAN subset, PAM-α1, to form sleep-independent memory. Data are mean ± SEM. ***p < 0.001 (Mann–Whitney *U* tests).

The data above indicated that PAM DAN subsets respond differentially to the presence or absence of food and thereby contribute to sleep-dependent or sleep-independent memory, respectively. Given that different sugars, based on their sweetness, induce different types of memory, we next asked whether these sugars differentially activate PAM DANs. Trained flies were kept on arabinose, sucralose, or sorbitol for 2 h following training, and then fly brains were imaged for calcium using CaLexA. We found that trained flies kept on arabinose or sucralose demonstrate a substantial increase in the GFP signal in PAM-β′2mp compared with flies moved to sorbitol (Fig. 6*A*, Mann–Whitney *U* tests, *p* (trained-arabinose vs trained-sorbitol) < 0.0001, *p* (trained-sucralose vs trained-sorbitol) = 0.0001; Table 1). Conversely, the calcium signal was considerably higher in PAM-α1 DANs in flies moved to sorbitol than in flies kept on either arabinose or sucralose (Fig. 6*B*, Mann–Whitney *U* tests, *p* (trained-arabinose vs trained-sorbitol) = 0.0003, *p* (trained-sucralose vs trained-sorbitol) < 0.0001; Table 1). Together, our results demonstrate that sweet taste triggers sleep-dependent memory consolidation by signaling to PAM-β′2mp DANs, while starvation drives the recruitment of PAM-α1 DANs to form sleep-independent memory.

## Discussion

Flies can form sleep-dependent and sleep-independent memory in an appetitive conditioning paradigm, but how they switch between these distinct forms of memory consolidation remains unexplored. In this study, we found that Gr64f GRNs-mediated sweet taste signal is essential for sleep-dependent memory. Sweet taste activates a specific subset of PAM DANs, PAM-β′2mp, for the consolidation of sleep-dependent memory (Fig. 6*C*). In contrast, in the absence of sweet stimulus, sleep becomes dispensable for memory consolidation and PAM-α1 DANs are recruited to form long-term memory (Fig. 6*C*). These findings indicate that the presence of reward can modulate the role of sleep in memory consolidation.

### Sweet taste influences the requirement of sleep for memory consolidation

Taste serves as an important modality in feeding decisions in *Drosophila* as perceived sweetness is used to estimate the carbohydrate content of food. Flies prefer sweet sugars over substances with a higher nutritive value indicating that taste determines initial feeding preferences (Stafford et al., 2012). Sweetness drives sugar ingestion during odor-reward pairing that is essential to form appetitive memories (Burke and Waddell, 2011; Fujita and Tanimura, 2011). Also, sweet taste tempers food-seeking behavior as flies on a sweet but non-nutritive medium show lower activity than starved flies (Yang et al., 2015). Therefore, the presence of sweet taste modifies behavior by providing a sensation of food.

Flies kept on food vials require sleep to form appetitive memories (Chouhan et al., 2021). As in the case of the other behaviors mentioned above, sweet taste appears to be sufficient to signal the presence of food and thereby trigger sleep-dependent memory formation. We found that flies kept on sucralose, a sweet but non-nutritive sugar, show an enhancement in post-training sleep and require sleep to consolidate long-term memory (Fig. 1). In contrast, flies on sorbitol, a tasteless but nutritive substance, form sleep-independent memory (Fig. 1). Also, disrupting the activity of sweet-sensing Gr64f^+^ neurons results in the formation of sleep-independent memory in fed flies, while stimulating Gr64f^+^ neurons in starved flies induces a switch to sleep-dependent memory consolidation (Figs. 2 and 3). While sweet taste may induce sleep-dependent memory, it is also possible that processes driving sleep-dependent and sleep-independent memory consolidation interact so as to be mutually exclusive. For instance, as starvation suppresses sleep, it may trigger sleep-independent memory, while sweet taste could drive sleep-dependent memory consolidation by releasing starvation-induced suppression of sleep. Neural connections between the two relevant circuits (described below) could mediate such interactions.

Sleep is typically thought to be induced under conditions of higher energy consumption; however, our data indicate that, at least for sleep-dependent memory consolidation, sweet taste rather than caloric intake serves as a signal for sleep. Sweet taste is not an accurate reporter for the nutritional value of food. Indeed, flies switch their preference from non-nutritive sweeteners to tasteless nutritive sugars over time in a two-choice assay (Dus et al., 2011; Stafford et al., 2012). Therefore, sweet taste can only provide a short-term impression of the presence of food, which might be sufficient to induce sleep-dependent consolidation as flies form sleep-dependent or sleep-independent memory in the first 4 h after training.

### Distinct subsets of PAM DANs mediate sleep-dependent and sleep-independent memory consolidation

The reward signal from PAM DANs during training is essential to form odor-reward associations (Burke et al., 2012; Liu et al., 2012). MB neurons are tiled by individual DAN terminals and the dendrites of corresponding MBONs to form 15 anatomically and functionally distinct compartments (Aso et al., 2014). PAM DANs largely innervate distinct compartments of the horizontal MB lobes (Aso et al., 2014). As per a previous study, a recurrent circuit that couples PAM-α1 DANs with the corresponding MBON in the α1 MB compartment mediates appetitive memory consolidation in starved flies (Ichinose et al., 2015). However, we found that PAM-α1 DANs are dispensable for memory consolidation in fed flies (Fig. 4). Instead, PAM DANs that innervate the β′2mp compartment of the MB are essential for long-term memory in fed flies (Fig. 4). Consistent with the findings that distinct PAM DANs mediate memory consolidation in fed versus starved states, the activity of PAM-β′2mp DANs was higher in trained and fed flies, while calcium in PAM-α1 DANs was significantly higher in trained and starved flies (Fig. 5). These results complement previous work in rats, humans, and flies demonstrating that discrete neural circuits mediate sleep-dependent and sleep-independent memory consolidation (Graves et al., 2003; Rauchs et al., 2004; van der Helm et al., 2011; Weber et al., 2014; Vorster and Born, 2015; Chouhan et al., 2021).

PAM-β′2mp DANs signal reward to form “safety-memory” of the unpaired odor during spaced aversive conditioning (Jacob and Waddell, 2020). Intriguingly, these neurons connect to MBONs implicated in appetitive memory consolidation under fed and starved conditions (Pavlowsky et al., 2018; Chouhan et al., 2021). PAM-β′2mp DANs receive input from MBON-γ2α′1 neurons, which are required for memory under fed conditions, and through axo-axonic projections they connect to the MBON-γ1Pedc neurons that are essential for memory under starved conditions (Aso et al., 2014; Li et al., 2020). We propose that MBON-γ2α′1 neurons engage PAM-β′2mp DAN-mediated reward signaling to establish a sleep-dependent memory trace. By contrast, an axo-axonal connection from MBON-γ1Pedc neurons may modulate neurotransmission in PAM-β′2mp DANs such that it allows PAM-α1 DANs to form sleep-independent memory. Future work can investigate how these discrete neural circuits interact to form sleep-dependent or sleep-independent memories.

### Sweet taste processing determines the role of sleep in memory consolidation

Sweet taste is perceived by a set of seven transmembrane receptors on the proboscis and tarsal segments of the leg (Clyne et al., 2000; Dahanukar et al., 2007; Jiao et al., 2008). The taste information is transmitted by GRNs that terminate in the suboesophageal ganglion (Thorne et al., 2004; Wang et al., 2004). Second-order neurons of the gustatory system send projections from the suboesophageal ganglion to the central brain for higher-order processing of taste information (Kain and Dahanukar, 2015; Talay et al., 2017). A subset of these projections terminates in the superior protocerebrum that also contains processes from DANs (Talay et al., 2017). Consistent with a connection between sweet-sensing neurons and DANs, an exposure to sucrose evokes a robust calcium response in PAM DANs (Liu et al., 2012; May et al., 2020). Therefore, sweet taste may signal reward by stimulating PAM DANs. Consistently, we found a significant calcium/GFP increase in PAM-β′2mp DANs in fed flies compared with starved flies (Fig. 5). Also, flies kept on arabinose or sucralose after training demonstrate a significant increase in PAM-β′2mp DAN activity compared with flies moved to sorbitol following training (Fig. 6). We suggest that the Gr64f^+^ GRNs mediated sweet taste signal acts in conjunction with the feedback from MBON-γ2α′1 neurons to recruit PAM-β′2mp DANs to form sleep-dependent memory.

Rewarding experiences modulate behavior to enhance survival. These experiences are preferentially replayed during sleep for better retention (Dag et al., 2019; Tamaki et al., 2020; Sterpenich et al., 2021). Indeed, memory consolidation involves neuronal reactivation in the mesolimbic dopaminergic reward system during sleep (Lansink et al., 2009; Perogamvros and Schwartz, 2012; Feld et al., 2014). Conversely, sleep loss modifies reactivity in the reward centers of the brain such that it induces reward-seeking behavior (Holm et al., 2009; Gujar et al., 2011). Our work suggests that the processing of sweet taste reward signals determines the necessity for sleep in memory consolidation. A general role for reward in determining the need for sleep is open to investigation.
